# Robust Power Optimization for Downlink Cloud Radio Access Networks with Physical Layer Security

**DOI:** 10.3390/e22020223

**Published:** 2020-02-17

**Authors:** Yijia Zhang, Ruiying Liu

**Affiliations:** 1 School of Information Science and Technology, Zhejiang Sci-Tech University, Hangzhou 310018, China; 2 College of Mechanical and Electrical Engineering, Jiaxing University, Jiaxing 314001, China; liuruiying@mail.zjxu.edu.cn

**Keywords:** C-RAN, CSI uncertainty, physical layer security, beamforming

## Abstract

Since the cloud radio access network (C-RAN) transmits information signals by jointly transmission, the multiple copies of information signals might be eavesdropped on. Therefore, this paper studies the resource allocation algorithm for secure energy optimization in a downlink C-RAN, via jointly designing base station (BS) mode, beamforming and artificial noise (AN) given imperfect channel state information (CSI) of information receivers (IRs) and eavesdrop receivers (ERs). The considered resource allocation design problem is formulated as a nonlinear programming problem of power minimization under the quality of service (QoS) for each IR, the power constraint for each BS, and the physical layer security (PLS) constraints for each ER. To solve this non-trivial problem, we first adopt smooth $\ell_{0}$-norm approximation and propose a general iterative difference of convex (IDC) algorithm with provable convergence for a difference of convex programming problem. Then, a three-stage algorithm is proposed to solve the original problem, which firstly apply the iterative difference of convex programming with semi-definite relaxation (SDR) technique to provide a roughly (approximately) sparse solution, and then improve the sparsity of the solutions using a deflation based post processing method. The effectiveness of the proposed algorithm is validated with extensive simulations for power minimization in secure downlink C-RANs.

## 1. Introduction

The cloud radio access network (C-RAN) [1,2] has been recognized as a promising paradigm for the fifth generation (5G) wireless network [3] in reducing both capital and operating expenditures. In C-RAN, the signal processing for filtering, modulate/demodulate, and detection are moved to the baseband unit (BBU) pool or central unit (CU), and all the low-powered base stations (BSs) are connected to the BBU pool through high capacity fibre fronthaul links or microwave wireless links [2]. Although joint transmission scheme can be adopted to boost the spectral efficiency, one particular technical challenge is the energy consumption due to the fluctuation of data traffic during day and night. Therefore, it is of great importance and interest to reduce the network power consumed by the BSs and the corresponding fronthaul links [4,5,6,7].

To achieve power efficient C-RAN, BS mode selection has been proved to be an promising paradigm to save energy [4,5,6,7,8]. In particular, the BSs with corresponding fronthaul links are switched into sleep mode under low traffic conditions. However, to provide guaranteed quality-of-service (QoS) requirements for each information receiver (IR), the transmit power consumption should be increased. Hence, the BSs mode selection and beamforming are jointly designed to reduce the power consumption [4,6,7,8,9]. Due to the broadcast properties of wireless channels, information signals may be eavesdropped by the eavesdrop receivers (ERs). Although traditional cryptographic technique may guarantee communication security in the application layer, it is not suitable in the current wireless network due to the computation improvement of ERs. The beamforming technique is propitious to align the information signals to the intended IRs; however, security transmission cannot be guaranteed when ERs are close to IRs or the channel gains of ERs are large enough. As an alternative, the concept of physical layer security (PLS) can perfectly guarantee wireless information transmission against eavesdropping in an information-theoretic perspective [10,11,12,13]. When artificial noise (AN) [13] is introduced to resist eavesdropping, the performance gain can be obtained. In summary, the joint design of beamforming and AN have been widely investigated to achieve secure information transmission over the past few years [14,15,16]. In particular, the BS mode, beamforming, and AN are jointly designed in a secure downlink C-RAN for network power minimization.

Since dense low-powered BSs are deployed in C-RAN, the distances between BSs and IRs/ERs are decreased. As a result, the received signal-to-interference-plus-noise ratio (SINR) at each ER is strengthened. Moreover, the information signals can also be improved at each ER since the joint transmission provides many signal copies. Therefore, it is necessary to investigate the power consumption in C-RAN by taking into consideration the PLS and BS mode selection. To achieve power efficient secure C-RAN, three problems must be addressed: (i) which BSs should be active? (ii) how much power should be transmitted by active BSs for data transmission of IRs? and (iii) how much AN should be generated by active BSs for interfering the ERs?

Practically, it is difficult to obtain the perfect CSI of IRs and ERs due to estimation and quantization errors, as well as Doppler frequency [17,18,19]. More importantly, the imperfection in CSI can significantly deteriorate the system performance [18,19,20]. A robust design approach needs to deal with the CSI uncertainty. Therefore, it is of great importance to jointly optimize the BS mode, beamforming, and AN to minimize the network power consumption while providing guaranteed QoS for each IR with PLS in a robust design manner.

In this paper, the joint design problem of BS mode selection, beamforming, and AN is formulated as an integer nonlinear non-convex programming problem subject to the constraints of the SINR at each IR, the maximum power constraint at each BS, and the maximum received SINR constraint at each ER with CSI uncertainty. The challenges of our work originate from both a combinatorial composite objective and the infinite number of non-convex quadratic constraints. The essential idea of addressing the infinite number of non-convex quadratic constraints is to apply the S-lemma [21], while the combinatorial composites for BS mode can be achieved by controlling the sparsity structure of the corresponding solutions. In particular, the authors in [4,6] propose group sparse beamforming (GSBF) approaches to minimize the network power consumption in a downlink C-RAN by jointly designing the active remote radio heads (RRHs) and beamforming. Reference [22] proposes group sparsity and relaxed integer programming algorithms to optimize power consumption in C-RAN considering both the uplink and downlink transmission. Moreover, a two-stage rank selection framework is proposed in [7] for the BS mode selection and user association optimization problem by successively determining the BS mode. Unfortunately, since our constraints take PLS into consideration, the algorithms in [4,6,7,22] are not applied to solve our problem. By reformulating the integer-related term in objective function using the $\ell_{0}$-norm, the $\ell_{1}/\ell_{2}$-norm algorithm in [7] can be modified to solve our problem only when the semi-definite relaxation (SDR) technique with S-lemma is used to tackle the non-convex quadratic PLS constraints. This $\ell_{1}/\ell_{2}$-norm algorithm switching as many BSs as possible into sleep mode provides sufficiently sparse BS mode; however, the transmit power consumption is increased in order to satisfy the QoS requirements. More importantly, using the SDR technique by dropping the rank one constraints, the tightness of the rank one relaxation should be investigated. In addition, the second order cone programming (SOCP) transformation [7] cannot be applied since the PLS constraints in our problem cannot be reformulated as second order cone (SOC) ones. On the other hand, some existing works focus on joint beamforming and AN design to maximize sum rate [13] or minimize transmit power [14]. However, AN based power allocation algorithms [13] for secrecy capacity maximization cannot be applicable to the power minimization problem in C-RAN. In conclusion, all the aforementioned algorithms cannot be applied to solve the considered problem directly. Consequently, the major contributions of this paper can be summarized as follows:

Firstly, we formulate the joint BS mode, beamforming and AN problem as an integer nonlinear non-convex programming problem, subject to IRs’ SINR requirements, transmit power constraints, and PLS constraints with CSI uncertainties. We reformulate the original problem using a sparse beamforming formulation. By approximating the non-convex $\ell_{0}$-norm using a smooth function, we propose a general iterative difference of convex (IDC) based algorithm to find a local optimal solution for the difference of convex (DC) programming problem.

Secondly, using the S-lemma in conjunction with SDR technique, we propose a three-stage algorithm to find the sparse structure of the active BSs. Specifically, the rough sparse solutions are obtained by using the IDC-based SDP algorithm in stage 1. In stage 2, we further improve the sparsity of the solution using a deflation based post processing method, and we solve a transmit power optimization problem in stage 3.

Finally, we validate the effectiveness of proposed algorithm through extensive simulations. The results suggest that our proposed algorithm can provide robust solutions, and the algorithm can converge to a local optimal solution rapidly. The proposed algorithm achieves comparable performance compares with the exhaustive search algorithm, and our algorithm significantly outperforms the algorithm without considering the BS mode selection.

The remainder of this paper is organized as follows. The system model and the problem formulation are presented in Section 2. In Section 3, we transform the problem into a difference of convex programming, and propose a general iterative difference of convex algorithm. In Section 4, by applying the SDR technique, we propose a three-stage algorithm. Simulation results of the proposed algorithm are presented in Section 5. Finally, the concluding remarks are given in Section 6. Table 1 summarizes the major symbols used for the rest of this paper.

## 2. System Model and Problem Formulation

### 2.1. System Model

#### 2.1.1. Signal Model

**Figure 1:**
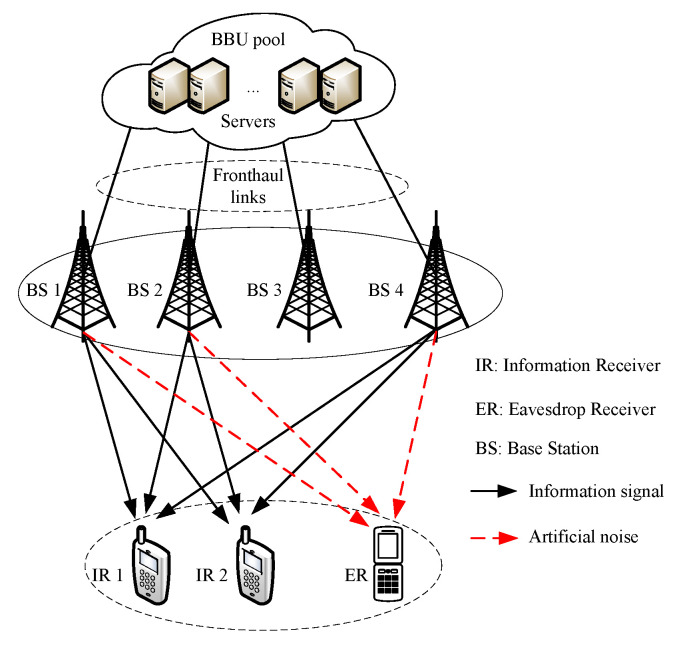
Illustration of the considered secure downlink C-RAN with L=4, K=2, M=1, A={BS1,BS2,BS4}.

We consider a secure downlink C-RAN consisting of *L* BSs (each equipped with *N* antennas), where the BSs jointly serve the *K* single-antenna IRs, which is illustrated in Figure 1. Denote L, A and *A* (A=|A|) the BSs set, active BS set, and the number of active BSs, respectively, and A⊆L. Note that there are *M* single-antenna ERs in the network.

The received signals at IR *k* (k∈{1,⋯,K}) and ER *m* (m∈{1,⋯,M}) are respectively given by

(1) $$y_{k}^{IR}=h_{k}^{H}x+n_{k}^{IR}\;\mathrm{and}\;y_{m}^{ER}=g_{m}^{H}x+n_{m}^{ER},$$

where $x\in C^{NA\times 1}$ denotes the joint transmit data vector of the *A* active BSs to the *K* IRs. The channels from the *A* active BSs to IR *k* and ER *m* are denoted by $h_{k}\in C^{NA\times 1}$ and $g_{m}\in C^{NA\times 1}$, respectively. $n_{k}^{IR}$ and $n_{m}^{ER}$ are respectively the noise at IR *k* and ER *m*, which are modeled as additive white Gaussian noise with zero mean and variances $\sigma_{IR_{k}}^{2}$ and $\sigma_{ER_{m}}^{2}$, respectively.

The transmitted signal $a_{k}$ with $E[|a_{k}{|}^{2}]=1$ for IR *k* is beamformed by $w_{k}$ before transmission. The beamformed signal for IR *k* is $x_{k}=w_{k}a_{k}$. The transmit signal vector x at the *A* active BSs is given by $x=\sum_{k=1}^{K}x_{k}+v$, where $v={\left[v_{1}^{T},\cdots ,v_{A}^{T}\right]}^{T}$ is the AN vector generated for the *A* active BSs ($v_{l}$ is the AN vector from BS *l* to IR *k*), which is modeled as a complex Gaussian random vector, i.e., v∼CN(0,V), where $V\in H^{NA}$, V⪰0 is the covariance matrix of v.

#### 2.1.2. Channel Model

The channel from the *A* BSs to the *k*-th IR is $h_{k}={\left[h_{k1}^{T},\cdots ,h_{kA}^{T}\right]}^{T}$. The downlink CSI of the BSs-to-IR channels can be obtained through measuring the uplink pilots in the handshaking or beacon signals via channel reciprocity. The BSs are able to refine the estimate of $h_{k}$ frequently via the pilot sequences embedded in each acknowledgement packet. Therefore, we assume that the channels from BSs to IR *k* are imperfectly known in the BBU pool, and the imperfect channel is modeled as the sum of two parts. According to [18,19], a deterministic model for characterizing the resulting uncertain channel is adopted

(2) $$h_{k}={\tilde{h}}_{k}+\Delta h_{k},\forall k,$$

where ${\tilde{h}}_{k}$ is the estimated channel of IR *k*, and $\Delta h_{k}={\left[\Delta h_{k1}^{T},\cdots ,\Delta h_{kA}^{T}\right]}^{T}\in C^{NA\times 1}$ is the channel uncertainty, which is assumed to satisfy an elliptic model, i.e., $\Delta h_{k}^{H}\Theta_{k}\Delta h_{k}\leq 1$ where $\Theta_{k}\in H^{NA\times NA}$ is the shape of the ellipsoid with $\Theta_{k}⪰0$. Define the set $H_{k}≜\left\{\Delta h_{k}:\Delta h_{k}^{H}\Theta_{k}\Delta h_{k}\leq 1\right\}$ which contains all possible channel uncertainty region of IR *k*.

The ERs do not interact with the BSs during information transmission, and the central unit does not know the location of ERs. However, to facilitate the resource allocation algorithm design, we follow the existing research [15] and the CSI between the BSs and the ERs are assumed to be known at central unit. As a result, we design the resource allocation algorithm assuming an unfavorable scenario (Without the instantaneous CSI of ERs at the central unit being known, stochastic geometry modeling may be an optimistic approach [23,24]. In this case, the system model is different from this paper, and we leave it for future work). In this paper, the same CSI model of ER is adopted as in [15,25]. Since the CSI of the ERs may be outdated during transmission, we use a deterministic model [17,18,19] for characterizing the resulting CSI uncertainty. The CSI model from BSs to ERs are

(3) $$g_{m}={\tilde{g}}_{m}+\Delta g_{m},\forall m,$$

where $g_{m}={\left[g_{m1}^{T},\cdots ,g_{mA}^{T}\right]}^{T}\in C^{NA\times 1}$, and $\Delta g_{m}={\left[\Delta g_{m1}^{T},\cdots ,\Delta g_{mA}^{T}\right]}^{T}\in C^{NA\times 1}$ is the channel uncertainty which is assumed to satisfy the following elliptic model (To model the imperfection CSI of ERs, a deterministic CSI error model [15,18,19] or probabilistic CSI error model [20] are usually adopted. The probabilistic CSI error model for ERs investigates the statistics of CSI. However, since the probabilistic CSI error model results in a highly intractable form, this paper only focuses on the deterministic CSI model), i.e., $\Delta g_{m}^{H}{\tilde{\Theta }}_{m}\Delta g_{m}\leq 1$, where ${\tilde{\Theta }}_{m}\in H^{NA\times NA}$ is the shape of the ellipsoid with ${\tilde{\Theta }}_{m}⪰0$. Define the set $\Omega_{m}≜\left\{\Delta g_{m}:\Delta g_{m}^{H}{\tilde{\Theta }}_{m}\Delta g_{m}\leq 1\right\}$ which contains all possible channel uncertainty region of ER *m*. It is noted that the spherical error model is considered when $\Theta_{k}=\frac{1}{\varepsilon_{IR_{k}}^{2}}I$ or ${\tilde{\Theta }}_{m}=\frac{1}{\varepsilon_{ER_{m}}^{2}}I$, where $\varepsilon_{IR_{k}}\geq 0$ and $\varepsilon_{ER_{m}}\geq 0$ are the radii of the uncertainty parts. In this case, the uncertainty vectors of IR and ER satisfy $∥\Delta h_{k}{∥}_{2}\leq \varepsilon_{IR_{k}}$ and $∥\Delta g_{m}{∥}_{2}\leq \varepsilon_{ER_{m}}$, respectively. It is easy to see that the CSI of both IR and ER become perfect when $\varepsilon_{IR_{k}}$ and $\varepsilon_{ER_{m}}$ approach zeros. Unlike considering the second-order statistics of channels [26], in this paper, we consider the instantaneous channel in every coherence interval. The beamforming vectors and AN, as well as BS mode are recomputed every coherence interval, and it can be viewed as an ideal or special case of the second-order statistics model.

The SINR at IR *k* and ER *m* are respectively

(4) $$\begin{array}{cc} {\mathrm{SINR}}_{k} & =\frac{|h_{k}^{H}w_{k}{|}^{2}}{\sum_{i\neq k}|h_{k}^{H}w_{i}{|}^{2}+{\left|h_{k}^{H}v\right|}^{2}+\sigma_{IR_{k}}^{2}},\forall k \end{array}$$

(5) $$\begin{array}{cc} {\mathrm{SINR}}_{m}^{k} & =\frac{|g_{m}^{H}w_{k}{|}^{2}}{\sum_{i\neq k}|g_{m}^{H}w_{i}{|}^{2}+{\left|vg_{m}^{H}\right|}^{2}+\sigma_{ER_{m}}^{2}}\\ \; & \overset{\left(a\right)}{\leq }\frac{|g_{m}^{H}w_{k}{|}^{2}}{|vg_{m}^{H}{|}^{2}+\sigma_{ER_{m}}^{2}}≜{\bar{\mathrm{SINR}}}_{m}^{k},\forall k,m, \end{array}$$

where (a) in Equation (5) constitutes an upper bound (The upper bound in Equation (5) is reasonable when all the other beamformed IRs’ signals (except for IR *k*) and the corresponding channels of the *m*-th ER are orthogonal) on the received SINR at ER *m* for decoding the information of IR *k* [15].

#### 2.1.3. Power Model

According to [1,27], the network power consumption of C-RAN consists of the power consumption by the BSs and fronthaul links. By moving the baseband processing into CU, a BS transceiver mainly comprises power amplifier, radio frequency module, direct-current (DC)–DC power supply, active cooling system and alternating current (AC)–DC main supply [27]. According to [27], $P_{l}^{\mathrm{bs}}$ can be modeled as typical linear function

(6) $$P_{l}^{\mathrm{bs}}=\left\{\begin{array}{ccc} P_{l}^{a,\mathrm{bs}}+\frac{1}{\eta_{l}}P_{l}^{\mathrm{tx}}, & \; & 0<P_{l}^{\mathrm{tx}}\leq P_{l}^{\max},\forall l\\ P_{l}^{s,\mathrm{bs}}, & \; & P_{l}^{\mathrm{tx}}=0,\forall l, \end{array}\right.$$

where $P_{l}^{\mathrm{tx}}=\sum_{k=1}^{K}∥w_{kl}{∥}_{2}^{2}+{∥v_{l}∥}_{2}^{2}$ is the transmit power, $P_{l}^{a,\mathrm{bs}}$ is the hardware power consumption of BS *l* in the active mode, and the constant $P_{l}^{s,\mathrm{bs}}$ (typically nonzero) is the power consumption of BS *l* in the sleep mode. Typically, since $P_{l}^{s,\mathrm{bs}}<P_{l}^{a,\mathrm{bs}}$, it is beneficial to switch BSs into the sleep mode to save energy. The static power for BS *l* is defined as $P_{l}^{c,\mathrm{bs}}=P_{l}^{a,\mathrm{bs}}-P_{l}^{s,\mathrm{bs}}$, which indicates the saved power of switching the BS from active into sleep mode, and we assume all the power amplifier has the same efficiency for simplicity, i.e., $\eta =\eta_{l},\forall l$.

According to [28], optical fiber is usually adopted by C-RAN as a fronthaul network to connect BSs and CU using a passive optical network (PON). The fronthaul network is comprised by an optical line terminal (OLT) that connects one optical fibre with a set of optical network unit (ONU). One efficient way to save energy for PON is to switch ONU into sleep mode. However, OLT cannot be switched into sleep mode and it consumes constant power. Therefore, the overall power consumed by fronthaul network is

(7) $$P^{\mathrm{fhn}}=P_{\mathrm{olt}}+\sum_{l=1}^{L}P_{l}^{\mathrm{tl}}$$

where $P_{\mathrm{olt}}$ is the constant power consumption of OLT, $P_{l}^{\mathrm{tl}}=P_{l}^{a,\mathrm{tl}}$ is the power consumption of ONU *l* in the active mode, and $P_{l}^{\mathrm{tl}}=P_{l}^{s,\mathrm{tl}}$ is the power consumption of ONU *l* in the sleep mode, and $P_{\mathrm{olt}}=20$ Watt, $P_{l}^{a,\mathrm{tl}}=3.85$ Watt and $P_{l}^{s,\mathrm{tl}}=0.75$ Watt. Therefore, switching off ONUs into sleep mode is an efficient way to save energy on the fronthaul network.

It is noted that, when both BS *l* and its corresponding fronthaul link *l* are in active mode, the power consumed by BS *l* and ONU *l* is $P_{l}^{a}=P_{l}^{a,\mathrm{tl}}+P_{l}^{a,\mathrm{bs}}$. We define $P_{l}^{s}=P_{l}^{s,\mathrm{tl}}+P_{l}^{s,\mathrm{bs}}$ the power consumption of BS *l* and ONU *l* in the sleep mode. Then, the network power consumption of C-RAN is

(8) $$\begin{array}{cc} P_{\mathrm{total}} & =\frac{1}{\eta }\sum_{l\in A}P_{l}^{\mathrm{tx}}+\sum_{l\in A}P_{l}^{a}+\sum_{l\in Z}P_{l}^{s}+P_{\mathrm{olt}}\\ \; & =\frac{1}{\eta }\sum_{l\in A}P_{l}^{\mathrm{tx}}+\sum_{l\in A}\left(P_{l}^{a}-P_{l}^{s}\right)+\sum_{l\in L}P_{l}^{s}+P_{\mathrm{olt}}\\ \; & =\frac{1}{\eta }\sum_{l\in A}\left(\sum_{k=1}^{K}∥w_{kl}{∥}_{2}^{2}+{∥v_{l}∥}_{2}^{2}\right)+\sum_{l\in A}P_{l}^{c}+\sum_{l\in L}P_{l}^{s}+P_{\mathrm{olt}}, \end{array}$$

where $P_{l}^{c}=P_{l}^{a}-P_{l}^{s}$ is the saved power by switching the BS *l* and ONU *l* into the sleep mode, and the second equality in (8) is based on the fact $\sum_{l\in Z}P_{l}^{s}=\sum_{l\in L}P_{l}^{s}-\sum_{l\in A}P_{l}^{s}$. Since this paper explores the power efficiency in secure downlink C-RAN, the constant term $\sum_{l\in L}P_{l}^{s}+P_{\mathrm{olt}}$ is omitted. Therefore, the network power (NP) consumption is given by

(9) $$P_{\mathrm{NP}}=\sum_{l\in A}\frac{1}{\eta_{l}}\left(\sum_{k=1}^{K}∥w_{kl}{∥}_{2}^{2}+{∥v_{l}∥}_{2}^{2}\right)+\sum_{l\in A}P_{l}^{c}.$$

It is noted that, switching off more BSs into sleep mode, more transmit power (summation of data transmission power (DTP) and AN) will be consumed in order to satisfy the QoS requirements. Therefore, the BS mode and transmit power should be jointly designed under certain QoS constraints.

### 2.2. Problem Formulation

To release the impact of limited-capacity fronthaul links on performance, fronthaul compression strategy is introduced in C-RAN. By introducing compression noise (or called quantized noise), the fronthaul capacity requirement is largely reduced. On the other hand, AN is introduced to interfere ERs to provide PLS. According to [29], compression noise on fronthaul links can be viewed as AN to provide PLS for C-RAN since they have the same mathematical form. Therefore, adding the fronthaul capacity constraints does not affect the convex form of the considered problem, and the solutions proposed in this paper can be directly extended to solve the problem with fronthaul compression under limited fronthaul capacity constraints.

We formulate the joint optimization problem of BS mode, AN and beamforming, subject to IR’s SINR requirements, transmit power constraints, and PLS constraints with CSI uncertainty, as a nonlinear non-convex programming problem, given by

(10a) $$\begin{array}{cc} P_{0}:\min_{w,v,A} & \;\frac{1}{\eta }\sum_{l\in A}\left(\sum_{k=1}^{K}∥w_{kl}{∥}_{2}^{2}+{∥v_{l}∥}_{2}^{2}\right)+\sum_{l\in A}P_{l}^{c} \end{array}$$

(10b) $$\begin{array}{cc} s.t. & \;\min_{\Delta h_{k}\in H_{k}}{\mathrm{SINR}}_{k}\geq \gamma_{k},\forall k, \end{array}$$

(10c) $$\begin{array}{cc} \; & \;\max_{\Delta g_{m}\in \Omega_{m}}{\bar{\mathrm{SINR}}}_{m}^{k}\leq \Gamma_{m}^{k},\forall k,m, \end{array}$$

(10d) $$\begin{array}{cc} \; & \;\sum_{k=1}^{K}∥w_{kl}{∥}_{2}^{2}+{∥v_{l}∥}_{2}^{2}\leq P_{l}^{\max},\forall l\in A, \end{array}$$

where $w=[w_{1},\cdots ,w_{K}]$. $\gamma_{k}$ is the SINR threshold of IR *k*, and constraints (10b) indicate that each IR has its minimum SINR requirement for a given channel uncertainty set $H_{k}$. $\Gamma_{m}^{k}$ is the SINR threshold of ER *m* for IR *k*, and constraints (10c) indicate that each ER has its maximum SINR requirement for a given channel uncertainty set $\Omega_{m}$. Constraint (10d) the power constraint for each BS with maximum transmit power $P_{l}^{\max}$. It is interesting to investigate the secrecy data rate maximization subject to the same constaints of problem $P_{0}$, and we leave it for future work.

The challenges of solving problem $P_{0}$ arise from: (i) the combinatorial objective function for BS selection; (ii) non-convex quadratic constraints (10b); and (iii) the infinite number of non-convex PLS constraints (10c). Even with convex constraints, the optimal solutions of problem $P_{0}$ are only achieved by exhaustive search procedure. Moreover, the computational complexity will exponentially increase with the network size. Therefore, this paper proposes a low-complexity algorithm based on DC procedure to find a local optimal solution to problem $P_{0}$.

## 3. DC-Based Sparse Beamforming Design

In this section, we first reformulate the above problem into a sparse beamforming one and convert the transformed problem to DC programs by using the smooth $\ell_{0}$-norm approximation. Then, we propose a DC-based algorithm to find a local optimal solution.

### 3.1. Problem Reformulation

In this subsection, we rewrite the combinatorial composite problem $P_{0}$ as a sparse beamforming form and approximate the $\ell_{0}$-norm using a smooth function.

#### 3.1.1. Sparse Beamforming Problem

It is observed that the mode of BSs can be specified with the beamforming w and v. In particular, when $\sum_{k=1}^{K}{∥w_{kl}∥}_{2}^{2}=0$ and $∥v_{l}{∥}_{2}^{2}=0$, BS *l* is switched into sleep mode, otherwise the BS should be active. By introducing a non-negative auxiliary variable $s_{l}=\sum_{k=1}^{K}∥w_{kl}{∥}_{2}^{2}+{∥v_{l}∥}_{2}^{2}$, we have

(11) $$f\left(s_{l}\right)=\left\{\begin{array}{ccc} 0, & \; & s_{l}=0,\forall l,\\ 1, & \; & \mathrm{otherwise}, \end{array}\right.$$

where $s_{l}$ can be viewed as the soft transmit power of BS *l*.

Since the $\ell_{0}$-norm indicates the number of nonzero elements of a vector, the indicator function $f(s_{l})$ can be replaced by the $\ell_{0}$-norm of $s_{l}$ without loss of optimality. Therefore, the original optimization problem $P_{0}$ is equivalently rewritten as

(12a) $$\begin{array}{cc} P_{1}:\min_{w,v,A,s} & \;\frac{1}{\eta }\sum_{l\in L}s_{l}+{∥s_{1}P_{l}^{c},\cdots ,s_{L}P_{L}^{c}∥}_{0} \end{array}$$

(12b) $$\begin{array}{cc} s.t. & \;\sum_{k=1}^{K}∥w_{kl}{∥}_{2}^{2}+{∥v_{l}∥}_{2}^{2}\leq s_{l},\forall l, \end{array}$$

(12c) $$\begin{array}{cc} \; & \;s_{l}\leq P_{l}^{\max},\forall l\in A,\\ \; & \;(10b),(10c), \end{array}$$

where $s={\left[s_{1},\cdots ,s_{L}\right]}^{T}$ and the sparsity of the BS mode is controlled by s. It is noted that, if the parameters of $\gamma_{k}$, $\Gamma_{m}^{k}$ and $P_{l}^{\max}$ are poorly chosen, problem $P_{1}$ may become infeasible. Then, user admission control or SINR relaxation should be applied, which is beyond the scope of this paper.

Although we have transformed problem $P_{0}$ to a sparse beamforming problem $P_{1}$, it is still challenging due to the non-convex $\ell_{0}$-norm in the objective, the non-convex quadratic QoS constraints and the infinite number of non-convex PLS constraints.

#### 3.1.2. Smooth $\ell_{0}$-Norm Approximation

To address the non-convex discontinuous $\ell_{0}$-norm in the objective, in this paper, we employ a general smooth function, denoted by f(x). The smooth function f(x) should satisfy the following three properties: (i) f(x) is concave and non-decreasing with respective to x≥0; (ii) $\lim_{x\to 0^{+}}f\left(x\right)=0$; and (iii) f(x) is continuous differentiable. Specifically, the logarithmic function, exponential function, and arctangent function are frequently adopted to approximate the non-convex $\ell_{0}$-norm [30,31], given by

(13) $$f_{\tau }\left(x\right)=\left\{\begin{array}{c} \frac{\log(1+x\tau^{-1})}{\log(1+\tau^{-1})},\\ 1-\exp(-x\tau^{-1}),\\ \arctan(x\tau^{-1}), \end{array}\right.$$

where τ>0 is used to control the smoothness of approximation. It is easily observed that a smoother function is obtained with a larger τ, but the approximation performance is worse, and vice versa.

Then, by replacing the non-convex $\ell_{0}$-norm in the objective with the smoothed function, the problem $P_{1}$ can be approximated as:

(14a) $$\begin{array}{cc} P_{2}:\min_{W,V,s} & \;\frac{1}{\eta }\sum_{l=1}^{L}s_{l}+\sum_{l=1}^{L}f_{\tau }\left(s_{l}\right)P_{l}^{c}\\ s.t. & \;(10b),(10c),(12b),(12c). \end{array}$$

Since the first term in the objective is convex in $s_{l}$ and the smooth function $f_{\tau }\left(s_{l}\right)$ is concave in $s_{l}$, the objective function is in a form of “a convex function + a concave function”. Therefore, the objective of problem $P_{2}$ is the difference between two convex functions. If the constraints in problem $P_{2}$ can be rewritten as the difference between two convex functions or be transformed to convex ones, problem $P_{2}$ is a general form of a DC programming problem. Then, the DC programming algorithm can be developed to deal with the DC programming problem. Therefore, an IDC procedure can be adopted to find a local optimal solution of problem $P_{2}$.

### 3.2. Generalized IDC Procedure

In this subsection, the IDC algorithm is proposed to solve problem $P_{2}$.

#### 3.2.1. IDC Procedure

The main idea for the IDC algorithm is to convexify the concave parts by their first order Taylor expansions, and then solve a sequence of convex problems successively. In particular, the objective function $f_{\tau }\left(s_{l}\right)$ is linearized, which is given by

(15) $$f_{\tau }\left(s_{l}^{\left(t\right)}\right)=f_{\tau }\left(s_{l}^{\left(t\right)}\right)+\nabla f_{\tau }\left(s_{l}^{\left(t-1\right)}\right)\left(s_{l}^{\left(t\right)}-s_{l}^{\left(t-1\right)}\right),$$

where t≥1 is the iteration number, and $f_{\tau }\left(s_{l}^{\left(t\right)}\right)$ is the value of $f_{\tau }\left(s_{l}\right)$ at the *t*-th iteration, and $\nabla f_{\tau }\left(s_{l}^{\left(t-1\right)}\right)$ is the gradient of $f_{\tau }\left(s_{l}\right)$ at the previous iteration point $s_{l}^{\left(t-1\right)}$.

Therefore, with the knowledge obtained from iteration (t−1), the following problem is solved at the *t*-th iteration

(16a) $$\begin{array}{cc} P_{3}:\min_{W,V,s} & \;\sum_{l=1}^{L}\nu_{l}s_{l}\\ s.t. & \;(10b),(10c),(12b),(12c), \end{array}$$

where the superscript (*t*) is dropped here, and $\nu_{l}=\frac{1}{\eta }+\nabla f_{\tau }\left(s_{l}^{\left(t-1\right)}\right)P_{l}^{c}$ is a constant.

Then, starting with an initial feasible point $s^{\left(0\right)}={\left[s_{1}^{\left(0\right)},\cdots ,s_{L}^{\left(0\right)}\right]}^{T}$, problem $P_{2}$ is iteratively solved through solving a sequence of problem $P_{3}$. Note that the smooth function $f_{\tau }\left(s_{l}\right)$ is strictly monotonic decreasing. Therefore, the IDC algorithm is guaranteed to converge to a local optimal solution of problem $P_{2}$, and the convergence proof can be found in [32,33]. Now, the main challenge of the IDC algorithm is to solve problem $P_{3}$. In the next subsection, the SDR technique with S-lemma is applied to solve problem $P_{3}$ efficiently.

#### 3.2.2. Updating Rule of τ

As mentioned in the above subsection, the approximation performance of $f_{\tau }\left(x\right)$ depends on the smoothed factor τ. Clearly, when *x* is small, τ should be small so that $f_{\tau }\left(x\right)$ approximate $\ell_{0}$-norm well, and τ should be large when *x* is large. As shown in [31], when τ is chosen to maximize the gradient of the approximation function, the three smooth functions in (13) have almost the same performance. Similar to the updating rule of τ in [31], we set a large value of τ for initialization, and decrease τ by a given factor ς, i.e., τ←ςτ. The τ is iteratively updated until it is sufficiently small.

It is noted that this general IDC algorithm can be applied to solve the unconstrained or linearly constrained problems [34]. Indeed, this algorithm is suitable for solving the general DC programming problems [35,36], for instance, a DC objective function with convex constraints or a convex objective function with DC constraints. However, the difficulties for the considered problem mainly come from the CSI uncertainty and PLS. Even with S-lemma and semi-definite relaxation technique, the rank one solutions still need to be investigated. In the next section, we will propose a three-stage algorithm by applying the IDC framework in conjunction with constraints transformations to solve the considered problem.

## 4. Proposed Optimization Algorithm

In this section, we develop a three-stage low-complexity algorithm with the SOCP transformation. Specifically, in stage 1, a rough sparse solution for the BS mode is obtained by applying the IDC-based SDP algorithm. In stage 2, a post processing procedure with a newly defined incentive metric is proposed to further improve the sparse structure of the solutions, and followed by optimizing the transmit power in stage 3.

### 4.1. Stage 1: IDC-Based SDP Algorithm

The challenge of the IDC procedure for solving problem $P_{2}$ is the non-convexity of problem $P_{3}$. While the non-convexity comes from the non-convex quadratic SINR constraints in constraints (10b) and the infinite number of PLS constraints (10c), which makes problem $P_{3}$ formidable to solve. Therefore, our focuses are to transform constraints (10b) and (10c) into tractable ones, and then recast problem $P_{3}$ into an SDP one.

To deal with the non-convex constraints (10b), we first transform it into an equivalent form, given by

(17) $$∥h_{k}^{H}w_{k}{∥}_{2}^{2}-\gamma_{k}\sum_{i\neq k}∥h_{k}^{H}w_{i}{∥}_{2}^{2}-{∥h_{k}^{H}v∥}_{2}^{2}\geq \gamma_{k}\sigma_{IR_{k}}^{2}.$$

Letting $W_{k}=w_{k}w_{k}^{H}$ and $X_{k}=W_{k}-\gamma_{k}\sum_{i\neq k}W_{i}-V$, we further rewrite constraint (17) as

(18) $${\left({\tilde{h}}_{k}+\Delta h_{k}\right)}^{H}X_{k}\left({\tilde{h}}_{k}+\Delta h_{k}\right)\geq \gamma_{k}\sigma_{IR_{k}}^{2},\forall \Delta h_{k}\in H_{k}.$$

It is found that constraint (18) refers to an infinite number of constraints because of the uncertainty in $h_{k}$. Towards this end, we transform (18) into linear matrix inequality (LMI) using the S-lemma [21]. Specifically, there exists $\Delta h_{k}$ satisfying the SINR constraints (18) for any $\Delta h_{k}^{H}\Theta_{k}\Delta h_{k}-1\leq 0$, if and only if there exists $x_{k}\geq 0,\forall k$, such that the following LMI constraints hold:

(19) $$\begin{array}{c} \left[\begin{array}{cc} X_{k} & X_{k}{\tilde{h}}_{k}\\ {\tilde{h}}_{k}^{H}X_{k} & {\tilde{h}}_{k}^{H}X_{k}{\tilde{h}}_{k}-\gamma_{k}\sigma_{IR_{k}}^{2} \end{array}\right]+x_{k}\left[\begin{array}{cc} \Theta_{k} & 0\\ 0 & -1 \end{array}\right]⪰0,\forall k, \end{array}$$

where $x=[x_{1},\cdots ,x_{K}]$ is the introduced variables for IRs.

Therefore, constraint (19) is a semi-definite. For constraints (10c), we first transform it into the following form

(20) $$\max_{\Delta g_{m}\in \Omega_{m}}\mathrm{Tr}\left(G_{m}W_{k}\right)/\Gamma -\mathrm{Tr}\left(G_{m}V\right)\leq \sigma_{ER_{m}}^{2},\forall k,m.$$

Following the similar transformation using the S-lemma, $\Delta g_{m}^{H}\Delta g_{m}\leq \sigma_{ER_{m}}^{2}$ implies

(21) $$\Delta g_{m}^{H}\left(\frac{W_{k}}{\Gamma }-V\right)\Delta g_{m}+2Re\left\{g_{m}^{H}\left(\frac{W_{k}}{\Gamma }-V\right)\Delta g_{m}\right\}+g_{m}^{H}\left(\frac{W_{k}}{\Gamma }-V\right)g_{m}-\sigma_{ER_{m}}^{2}\leq 0,\forall m,k,$$

holds if and only if there exist $\alpha_{m}^{k}\geq 0$, such that the following LMI constraints hold.

(22) $$\begin{array}{cc} S(W_{k},V,\alpha_{m}^{k})= & -U_{g_{m}}^{H}W_{k}U_{g_{m}}/\Gamma +U_{g_{m}}^{H}VU_{g_{m}}\\ \; & +\alpha_{m}^{k}T_{m}^{k}+{\tilde{T}}_{m}⪰0,\forall m,k, \end{array}$$

where $T_{m}^{k}=\left[\begin{array}{cc} {\tilde{\Theta }}_{m} & 0_{NL\times 1}\\ 0 & -1 \end{array}\right]$ and ${\tilde{T}}_{m}=\left[\begin{array}{cc} 0_{NL} & 0_{NL\times 1}\\ 0 & \sigma_{ER_{m}}^{2} \end{array}\right]$, and $U_{g_{m}}=\left[I_{NL}\;{\tilde{g}}_{m}\right]$.

Hence, by directly dropping the rank one constraints, $\mathrm{rank}(W_{k})=1,\forall k$ and rank(V)=1, problem $P_{3}$ can be transformed as

(23a) $$\begin{array}{cc} P_{4}:\min_{W,V,s,x,\alpha } & \;\sum_{l=1}^{L}\nu_{l}s_{l} \end{array}$$

(23b) $$\begin{array}{cc} s.t. & \;\sum_{k=1}^{K}\mathrm{Tr}\left(\Phi_{l}W_{k}\right)+\mathrm{Tr}\left(\Phi_{l}V\right)\leq s_{l},\forall l\in L \end{array}$$

(23c) $$\begin{array}{cc} \; & \;s_{l}\leq P_{l}^{\max},\forall l\in L \end{array}$$

(23d) $$\begin{array}{cc} \; & \;V⪰0,W_{k}⪰0,\alpha_{m}^{k}\geq 0,\forall k,m\\ \; & \;(19)\;\mathrm{and}\;\left(22\right), \end{array}$$

where $\Phi_{l}≜\mathrm{diag}\left(\underset{(l-1)N}{\underset{︸}{0,\cdots ,0}},\underset{N}{\underset{︸}{1,\cdots ,1}},\underset{(L-l)N}{\underset{︸}{0,\cdots ,0}}\right)$ for all l∈L, $W=\{W_{k}\}$ and $\alpha =\{\alpha_{m}^{k}\}$.

Since problem $P_{4}$ is an SDP problem, we can solve it using the standard convex optimization software, such as CVX [37]. It is noted from the following proposition that the solution $s_{l}$ of problem $P_{4}$ cannot equal to zero, which means that the sparse structure of active BSs cannot be obtained.

**Proposition** **1.** *The solution of problem $P_{4}$ can be expressed as $s_{l}^{★}=\sum_{k=1}^{K}Tr\left(\Phi_{l}W_{k}^{★}\right)+Tr\left(\Phi_{l}V^{★}\right)$, where $W_{k}^{★}$ and $V^{★}$ are respectively the optimal solutions of $W_{k}$ and V, and $W_{k}^{★}$ cannot be zero matrices. The proof can be found in Appendix A.*

To illustrate the results in Proposition 1, we will give an experimental example in Section 4. Thus, the sparse solution of problem $P_{4}$ is generated by iteratively penalizing the BS with smaller transmit power consumption.

**Proposition** **2.** *The worst-case SINR constraints of IRs and the SINR constraints of ERs in problem $P_{4}$ should be active at the optimal solution.*

**Proof.**  Since constraints (19) and (22) are respectively the equivalent transformation of (10b) and (10c), we prove (10b) and (10c) are active at the optimal solution. Firstly, we prove that constraints (10b) are active at the optimal solution. If the left-hand side of (10b) is greater than γ, one can decrease DTP to save power. Since constraints (10c) must be satisfied, decreasing DTP does not affect the inequality. Thus, constraints (10b) must be active at the optimal solution. The first part proof of this proposition is completed.

Now, we prove the second part of this proposition. If Γ is greater than the left-hand side of (10c), one can decrease DTP or increase AN to save power. However, with the decrease of DTP or increase of AN, the left-hand side of (10b) is decreased. Then, the equality of (10b) is not satisfied, which conflicts with the equality constraints in (10b). The second part of the proof is completed. □

Since the rank one constraints are dropped in problem $P_{4}$, it is important to investigate tightness of such a relaxation. If the solutions of problem $P_{4}$ ($W_{k}^{★}$) are rank one, i.e., rank($W_{k}^{★}$) = 1, the optimal beamforming $w_{k}^{★}$ of problem $P_{4}$ can be extracted by eigenvalue decomposition from $W_{k}^{★}$. Otherwise, the Gaussian randomization method [38] can be employed to obtain the approximate solutions.

Finally, the algorithm is summarized in Algorithm 1.
**Algorithm 1** IDC-based SDP algorithm. **Initialization:** Set the initial values for $s^{\left(0\right)}$ by letting $\left\{W_{k}^{\left(0\right)},V^{\left(0\right)}\right\}$ be a feasible point to problem $P_{4}$; **Repeat:** **Step 1:** Fix $\left\{\nu_{l}\right\}$, solve problem $P_{4}$ and return instantaneous optimal solution $\left\{W_{k}^{★},V^{★},s^{★}\right\}$; **Step 2:** Update $\left\{W_{k}\leftarrow W_{k}^{★},V\leftarrow V^{★},s\leftarrow s^{★},\tau \leftarrow \varsigma \tau \right\}$, update $\left\{\nu_{l}\right\}$ using $s^{\left(0\right)}={\left[s_{1}^{★},\cdots ,s_{L}^{★}\right]}^{T}$. **Until** convergence or maximum iteration is reached.

Although the active BSs are obtained from Algorithm 1 by checking the nonzero elements of $s^{★}$, the minimum network power consumption may not be attained since Algorithm 1 converges to a local optimal solution. Since the IDC-based SDP algorithm tries to switch maximum number of BSs into sleep mode, the minimum network power consumption cannot be attained. Moreover, as mentioned in [4], by investigating the system parameters after obtaining the beamforming and AN, the performance can be enhanced. Therefore, we develop a post processing procedure to further reduce the network power consumption in the next subsection.

### 4.2. Stage 2: Post Processing Procedure

The IDC-based SDP algorithm tries to enforce $s^{★}$ to zero, and the BSs with zero soft transmit power are switched into sleep mode. For instance, when $s_{l}^{★}=0$, BS *l* is switched into sleep mode. Since Algorithm 1 converges to a local optimal solution, using only $s_{l}^{★}$ to find the active BSs will cause a performance loss. However, the amount of soft transmit power indicates the BS activation priority, and a smaller $s_{l}^{★}$ indicates that BS *l* has a lower priority to be switched off. According to [4,7], a better network power performance can be obtained by exploiting the key system parameters. Hence, in this subsection, we define a new incentive metric $\Lambda_{l}$ to indicate the priority of activating BS *l* as

(24) $$\begin{array}{c} \Lambda_{l}=\frac{s_{l}^{★}\sum_{k=1}^{K}{∥h_{kl}∥}_{2}^{2}}{P_{l}^{c}}, \end{array}$$

where $\sum_{k=1}^{K}{∥h_{kl}∥}_{2}^{2}$ indicates the channel gain of BS *l*. A larger $\Lambda_{l}$ means that BS *l* has a higher priority to be activated.

It should be noted that the BSs with no transmit power (i.e., $s_{l}^{★}=0$) should be switched off since the corresponding $\Lambda_{l}$ equals to zero when $s_{l}^{★}=0$. Thus, $Z^{\left[0\right]}=\left\{s_{l}^{★}=0\right\}$ denotes the roughly inactive BS set. Denote $\hat{A}=L\setminus \left\{s_{l}^{★}=0\right\}$ the rough active BS set obtained from Algorithm 1, and $\hat{A}=\hat{A}$ the rough number of active BSs. Then, all the incentive metric are sorted in ascending order: $\theta_{\pi_{1}}\leq \cdots \leq \theta_{\pi_{\hat{A}}}$, where $\pi_{i}$ is the active BSs order. By fixing the active BS set $A^{\left[i\right]}$ at the *i*-th step, problem $P_{4}$ becomes

(25) $$\begin{array}{cc} P_{5}:\min_{W,V,x,\alpha } & \;\frac{1}{\eta }\sum_{l\in A^{\left[i\right]}}\left(\sum_{k=1}^{K}\mathrm{Tr}\left(\Phi_{l}W_{k}\right)+\mathrm{Tr}\left(\Phi_{l}V\right)\right)+\sum_{l\in A^{\left[i\right]}}P_{l}^{c}\\ s.t. & \;\sum_{k=1}^{K}\mathrm{Tr}\left(\Phi_{l}W_{k}\right)+\mathrm{Tr}\left(\Phi_{l}V\right)\leq P_{l}^{\max},\forall l\in A^{\left[i\right]}\\ \; & \;\left(19),(22),(23d\right), \end{array}$$

The post processing procedure switches the BSs with the lowest incentive metric into sleep mode one by one until problem $P_{5}$ becomes infeasible or the network power consumption of problem $P_{5}$ at the *i*-th step is larger than the power consumption obtained from the previous [i−1]-th step, where $A^{\left[i\right]}$ is the instantaneous active BS set at the *i*-th step, and $A^{\left[i\right]}=L\setminus Z^{\left[i\right]}$ with $Z^{\left[i\right]}=\left\{\pi_{1},\cdots ,\pi_{i}\right\}$.

Since the BSs are switched into sleep mode one by one, problem $P_{5}$ needs to solve by no more than $\hat{A}$ times. Moreover, since problem $P_{5}$ has a similar form as problem $P_{4}$, the rank one conditions are also attainable, which can be easily proved.

### 4.3. Stage 3: Transmit Power Optimization

Once the active BS set $A^{★}$ is obtained using Algorithm 2, the joint beamforming and AN design problem needs to be solved. In other words, by replacing $A^{\left[i\right]}$ with $A^{★}$, we solve the following problem:

(26) $$\begin{array}{cc} P_{6}:\min_{W,V,\alpha } & \;\frac{1}{\eta }\sum_{l\in A^{★}}\left(\sum_{k=1}^{K}\mathrm{Tr}\left(\Phi_{l}W_{k}\right)+\mathrm{Tr}\left(\Phi_{l}V\right)\right)+{\hat{A}}^{★}P_{l}^{c}\\ s.t. & \;\sum_{k=1}^{K}\mathrm{Tr}\left(\Phi_{l}W_{k}\right)+\mathrm{Tr}\left(\Phi_{l}V\right)\leq P_{l}^{\max},\forall l\in A^{★}\\ \; & \;\left(19),(22),(23d\right), \end{array}$$

where ${\hat{A}}^{★}=\left|A^{★}\right|$ is the active number of active BSs, and problem $P_{6}$ is a SDP problem which can be solved using the interior-point method [21]. It is obvious that problem $P_{6}$ has rank one solutions.

Finally, the post processing is summarized as Algorithm 2.
**Algorithm 2** Post processing procedure for finding the final active BSs. **Initialization:** let $A^{\left[0\right]}=\left\{1,\cdots ,\hat{A}\right\}$, the network power consumption $P_{\mathrm{total}}^{A^{\left[0\right]}}$ and the iteration index i=0. **Step 1:** Update the iteration number i=i+1. **Step 2:** Let $A^{\left[i\right]}=A^{\left[i-1\right]}\setminus \left\{\pi_{i}\right\}$, and solve problem $P_{5}$. **Step 3:** If problem $P_{5}$ is infeasible, go to **Step 5**; Otherwise, go to **Step 4**. **Step 4:** If $P_{\mathrm{total}}^{A^{\left[i\right]}}\leq P_{\mathrm{total}}^{A^{\left[i-1\right]}}$, go to **Step 1**; Otherwise, go to **Step 5**. **Step 5:** Obtain the final active BS set $A^{★}=A^{\left[i-1\right]}$. **Step 6:** Solve problem $P_{6}$ and obtain the final network power consumption $P_{\mathrm{total}}^{A^{★}}$, beamforming and AN (i.e., $\left\{W_{k}^{★},V^{★}\right\}$).

### 4.4. Initialization and Complexity Analysis

In this subsection, we first discuss the initial points for Algorithm 1, and then analyze the computational complexity of the proposed algorithm.

#### 4.4.1. Initialization

Since Algorithm 1 needs to solve problem $P_{4}$ iteratively, it is important to set the starting point to be feasible. In this paper, we get a feasible point through solving the following initialization problem with all BSs active (i.e., let A=L).

(27) $$\begin{array}{cc} P_{\mathrm{INI}}:\min_{W,V,x,\alpha } & \;\frac{1}{\eta }\sum_{l\in L}\left(\sum_{k=1}^{K}\mathrm{Tr}\left(\Phi_{l}W_{k}\right)+\mathrm{Tr}\left(\Phi_{l}V\right)\right)\\ s.t. & \;\sum_{k=1}^{K}\mathrm{Tr}\left(\Phi_{l}W_{k}\right)+\mathrm{Tr}\left(\Phi_{l}V\right)\leq P_{l}^{\max},\forall l\in L\\ \; & \;\left(19),(22),(23d\right), \end{array}$$

The optimal solution $\left\{W_{k}^{\left(0\right)},V^{\left(0\right)}\right\}$ of problem $P_{\mathrm{INI}}$ is employed as a feasible initialization point in Algorithm 1 in Section 4.1. Then, the initialization point $s^{\left(0\right)}$ is calculated using $s_{l}^{\left(0\right)}=\sum_{k=1}^{K}\mathrm{Tr}\left(\Phi_{l}W_{k}^{\left(0\right)}\right)+\mathrm{Tr}\left(\Phi_{l}V^{\left(0\right)}\right),\forall l$. Since Algorithm 1 iteratively generates a decreasing sequence, it converges to a local optimal solution [32,33] with a initialization point $s^{\left(0\right)}$.

#### 4.4.2. Complexity Analysis

According to [39], the computational complexity of Algorithm 1 using the interior point method is on the order of $O(I_{\max}\sqrt{MKNL}\left(MK^{3}{\left(NL\right)}^{2}+MK^{2}{\left(NL\right)}^{3}\right))$, where $I_{\max}$ is the maximum iteration number. Since Algorithm 2 solves problem $P_{5}$ for at most $\hat{A}$ times and problem $P_{6}$ for one time, its computational complexity is $O(\hat{A}\sqrt{MKN\hat{A}}\left(MK^{3}{\left(N\hat{A}\right)}^{2}+MK^{2}{\left(N\hat{A}\right)}^{3}\right))$. Finally, the total computational complexity of the proposed algorithm (summation of Algorithms 1 and 2) is $O(\hat{A}\sqrt{MKN\hat{A}}\left(MK^{3}{\left(N\hat{A}\right)}^{2}+MK^{2}{\left(N\hat{A}\right)}^{3}\right))$.

## 5. Simulation Results and Discussion

**Figure 2:**
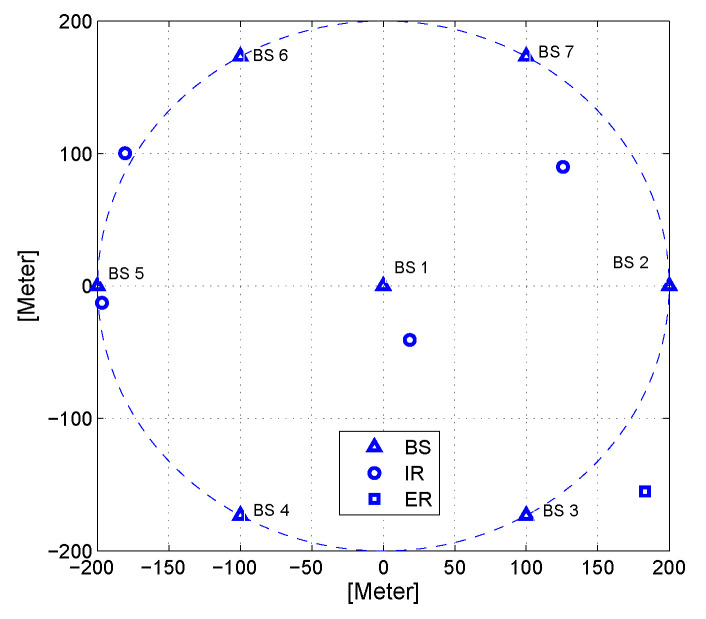
Layout of the secure downlink C-RAN.

We consider a secure downlink C-RAN with L=7 two-antenna BSs, K=4 single-antenna IRs and M=1 single-antenna ER. One BS locates at the circle centre, and the other six BSs are located on the circle rim with a radius of 0.2 km. The IRs and ER are randomly distributed in the circle with uniform distribution. The layout of the considered scenario in a secure downlink C-RAN is depicted in Figure 2.

We assume that all the BSs have the same fronthaul link power ($P^{c}=P_{l}^{c},\forall l$) and maximum transmit power ($P^{\max}=P_{l}^{\max},\forall l$). We set $P_{l}^{c}=5$ Watt and $P_{l}^{\max}=1$ Watt for all BSs [4]. The channel model considered in this paper consists of path-loss, shadowing and small-scale fading. In particular, the channel from BS *l* to IR *k* is

(28) $$\begin{array}{c} {\tilde{h}}_{kl}=10^{L(d_{kl})/20}\sqrt{\varphi_{kl}\delta_{kl}}b_{kl}, \end{array}$$

where $L\left(d_{kl}\right)=148.1+37.6\log_{10}d_{kl}$ is the path-loss fading from BS *l* to IR *k* at $d_{kl}$ km [4,40], $\varphi_{kl}=9$ dBi is the transmit antenna gain at each BS, $\delta_{kl}$ is the log-normal shadowing and zero mean and standard deviation of 8 dB of $\delta_{kl}$ are adopted in this paper [4], and $b_{kl}∼\mathrm{CN}\left(0,I\right)$ is the small-scale fading coefficient. Similarly, the channels (${\tilde{g}}_{m},\forall m\in M$) from BSs to ERs are generated. The channel uncertainties of IRs and ERs are assumed to satisfy $\Theta_{k}=\frac{1}{\varepsilon_{IR}^{2}}I_{NL},\forall k$ [19] and ${\tilde{\Theta }}_{m}=\frac{1}{\varepsilon_{ER}^{2}}I_{NL},\forall m$ [15], respectively, and without specified $\varepsilon_{IR}^{2}=\varepsilon_{ER}^{2}=0.05$. We default Γ=−10 dB [15] and $\gamma =\gamma_{k}=15$ dB. The noise power (with 10 MHz bandwidth) is $\sigma_{IR_{k}}^{2}=\sigma_{ER_{m}}^{2}=-104$ dBm. We utilize the general smooth function as $f\left(s_{l}\right)=\frac{\ln(1+s_{l}\tau^{-1})}{\ln(1+\tau^{-1})}$ [31]. Without specified, all the data are averaged over 40 independently IR and ER locations, and the CSI on each location is averaged over 40 channel realizations. Three algorithms are taken into consideration for comparison.

**$\ell_{1}$/$\ell_{2}$-norm algorithm [7]:** this algorithm is also called re-weighted $\ell_{1}$-norm algorithm, which approximates $\ell_{0}$-norm in the objective function of problem $P_{0}$ by the re-weighted $\ell_{1}$/$\ell_{2}$-norm, and updates the weights iteratively until the convergence condition is met. The BSs with zero transmit power consumption will be switched into sleep mode. Since the $\ell_{1}$/$\ell_{2}$-norm algorithm needs to solve a series of SDP problems and its computational complexity is in the order of $O(I_{\max}\sqrt{MKNL}\left(MK^{3}{\left(NL\right)}^{2}+MK^{2}{\left(NL\right)}^{3}\right))$, where $I_{\max}$ is the maximum iteration number.

**ExSearch algorithm:** this algorithm is the optimal one since it computes network power consumption for all possible combinations of the active BSs, and chooses the one with lowest power consumption. The corresponding BSs are active, and this algorithm achieves global optimal solution since the problem is globally solved for any fixed active BS mode. Since the Exsearch algorithm searches for all possible combinations of the active BSs, the computational complexity is on the order of $O(2^{L}\sqrt{MKNL}\left(MK^{3}{\left(NL\right)}^{2}+MK^{2}{\left(NL\right)}^{3}\right))$.

**Baseline algorithm:** this algorithm assumes that all the BSs are active. This algorithm is taken for comparison in order to investigate the performance gain of BS mode selection. ExSearch algorithm can be regarded as a special case of Baseline algorithm if the obtained active BS set $A^{★}$ of ExSearch algorithm is L. The Baseline algorithm solves the SDP problem for only one time and its computational complexity is in the order of $O(\sqrt{MKNL}\left(MK^{3}{\left(NL\right)}^{2}+MK^{2}{\left(NL\right)}^{3}\right))$.

### 5.1. Power Distribution of the BSs

In this subsection, we investigate the power distribution of the BSs with all BSs active to show the efficiency of proposition 1. Letting A=L (all BSs are active), we solve problem $P_{5}$ using mathematical tool, i.e., CVX. It is noted that problem $P_{5}$ is a power minimization problem without considering BS mode selection, and the BS power distribution will give some insights into which BSs are active when BS mode selection is taken into consideration. Since the fronthaul link power consumption is fixed, we only analyze the transmit power consumption of each BS for eight typical randomly channel realizations, and the results are listed in Table 2. Note that we use “Ch” as the abbreviation of “Channel”.

As shown in Table 2 that the transmit power of each BS is not equal to zero for all the eight random channel realizations. In fact, we have tested the transmit power distribution of the BS mode for more than 1000 randomly channel realizations, and found that the transmit power of the BSs is not equal to zero exactly. In other words, the value of dual variable $\beta_{l}$ is no less than 1/η in general. However, it is interesting to find that the transmit powers of some BSs are close to zero compared to other ones. For instance, BS 2, 3 and 4 are lower transmit power than that of the other four BSs for Channel 1, and BS 2, 4, and 7 also consume lower transmit than that of the other four BSs. It is also observed from Table 1 that BS 4 seems to be switched into sleep mode for Channel 7 since it consumes much lower transmit power than other BSs. The value of the transmit power provides some insights in selecting which BSs should be switched into sleep mode in the following subsections.

### 5.2. Convergence Analysis

**Figure 3:**
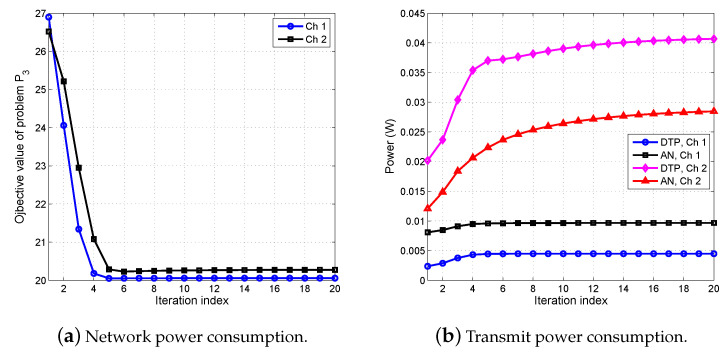
Convergence behavior of the SDR-based SDP algorithm for two random channels.

To demonstrate the convergence rate of the proposed SDR-based DC algorithm, we plot the objective value of problem $P_{3}$ over two randomly channel realizations. It is shown in Figure 3a that the proposed algorithm converges to a local optimal solution in less than 15 iterations. In Figure 3b, we show the corresponding DTP and AN distribution of Algorithm 1. It indicates that both DTP and AN increase with the iteration progress, and Algorithm 1 converges when DTP and AN converge to a local optimal solution. This is mainly due to the fact that Algorithm 1 tries to switch the BSs into sleep mode with the updated weights, and the transmit power must be increased in order to satisfy the same QoS requirements and PLS constraints. According to the expressions of the SINR of IRs and ERs, by increasing DTP, the SINRs of IRs increase while the SINRs of ERs decrease. On the other hand, the directions of transmitted signals try to align the intended IRs, and a small amount of AN is needed to interfere the ERs. As a result, the power consumption for data transmitting is larger than AN (AN accounts for only 19.5 % and 13.85 % of overall transmit power for Ch 1 and Ch 2, respectively).

**Figure 4:**
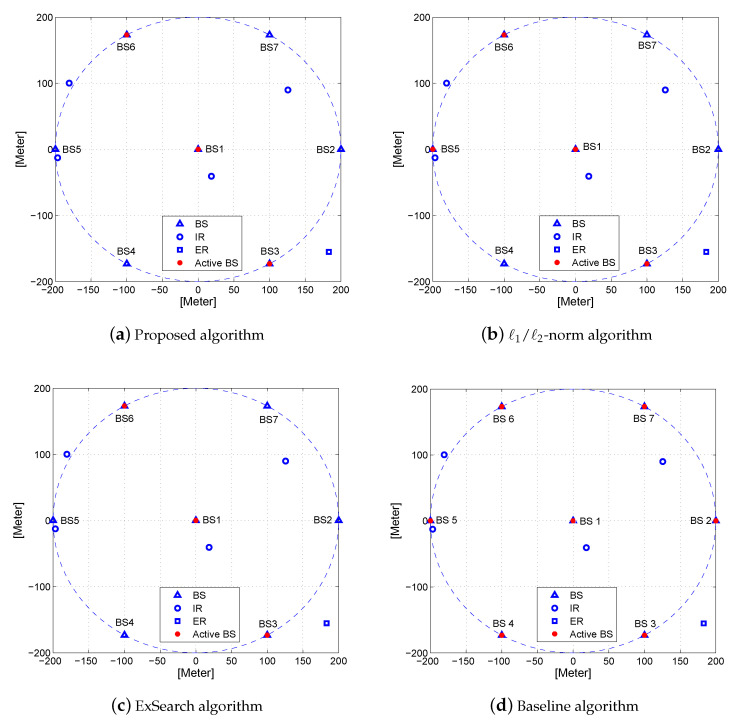
The set of active BSs generated by (**a**) proposed algorithm, (**b**) $\ell_{1}/\ell_{2}$-norm algorithm, (**c**) ExSearch algorithm, and (**d**) Baseline algorithm.

To investigate further, we take the active BSs of Ch 2 (the same channel as in Figure 3) under different algorithms as an example, which is given in Figure 4. It is observed from Figure 4 that $\ell_{1}/\ell_{2}$-norm algorithm needs four active BSs in order to support the required SINR of IRs and PLS constraints. However, the proposed three-stage algorithm consumes only three active BSs, which has the same active number as the ExSearch algorithm. This is mainly because the power consumption increased by the transmit power is lower than the power saved by switching one more BS into sleep mode while satisfying the same constraints. These observations reveal that the BSs with lower transmit power are inclined to be switched into sleep mode. The active BS number, network power, and transmit power consumption of the proposed three-stage algorithm are given in Table 3. Since all BSs are assumed to be active for the baseline algorithm, there are seven active BSs in Table 3. It can be seen from Table 3 that the proposed algorithm and ExSearch algorithm consume a smaller amount of network power than that of $\ell_{1}/\ell_{2}$-norm algorithm and the baseline algorithm.

### 5.3. Impact of SINR Threshold of IR on Performance

**Figure 5:**
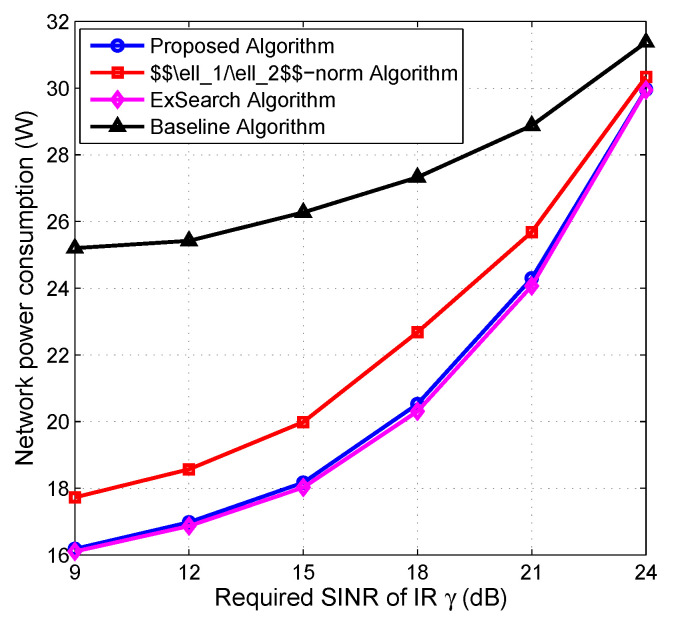
Network power consumption vs. minimum required SINR of IRs.

In Figure 5, we evaluate the impact of the minimum required SINR (SINR threshold) of IRs on the overall network power consumption. It can be observed that the network powers of all the algorithms are a monotonically nondecreasing function of γ. This is mainly because more active BSs and transmit power are consumed in order to support higher required SINR of IRs. The network power consumption of the proposed algorithm is close to the ExSearch one, and is lower than the baseline and $\ell_{1}/\ell_{2}$-norm algorithms by respectively 35% and 8% in the low required SINR region, and by about 12% and 5%, respectively, in the high required SINR region.

### 5.4. Impact of SINR Threshold of ER on Performance

**Figure 6:**
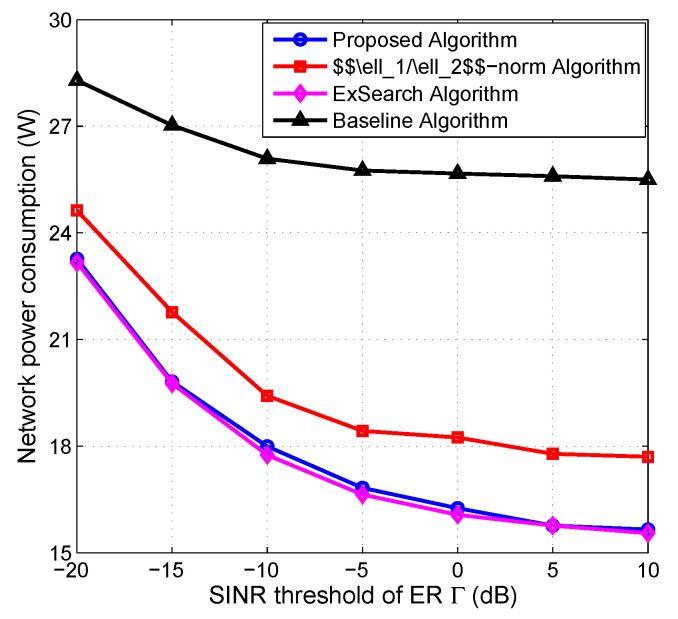
Network power consumption vs. maximum tolerate received SINR of ERs.

Figure 6 studies the impact of the ERs’ SINR threshold Γ on the network power consumption. It is observed from Figure 6 that a smaller Γ results in a higher overall power consumption. This is because more AN has to be generated to satisfy a smaller Γ, bringing in a larger interference. As a result, more DTPs are consumed to satisfy the minimum required SINR of IRs, resulting in a higher overall network power consumption. Moreover, the performance of the proposed algorithm is approximately the optimal one, and it consumes around 5% less network power than the $\ell_{1}/\ell_{2}$-norm algorithm.

### 5.5. Performance of the Proposed Algorithm

In this subsection, we investigate the relationship between network power consumption and ER number under different channel errors of IRs and ERs. All the IRs and ERs are randomly distributed in the circle region. For each IR and ER location, a single channel realization at each location is considered. Since the proposed algorithm has comparable network performance as the ExSearch one, and both achieve much better performance than $\ell_{1}/\ell_{2}$-norm algorithm and Baseline algorithm, we only plot the proposed algorithm in this subsection. In the simulations, we assume that all the IRs and ERs have the same CSI error radiuses, i.e., $\varepsilon^{2}=\varepsilon_{IR}^{2}=\varepsilon_{ER}^{2}$.

#### 5.5.1. Impact of BS Antennas on Performance

Firstly, we compare the performance of different algorithms under different BS antennas, and the results are given in Table 4. Intuitively, the network power consumption decreases with the increased BS antennas. More BS antennas provide a larger diversity gain, and less active BSs as well as transmit power are needed to guarantee QoS requirements and PLS. As can also be seen from Table 4, the total power consumption decrease with the increased number of BS antennas. With more BS antennas, degree of freedom provided by BSs is increased and the desired signals are concentrated to align IRs. As a result, less amount of AN is needed to provide PLS.

#### 5.5.2. Impact of CSI Error Radius of ERs on Performance

To investigate further, we study different CSI error radius of IRs and ERs on the performance of the proposed algorithm. The results are given in Table 5. As can be seen from Table 5 that both the transmit power and network power are increased with the increasing CSI error radius of ERs. This is due to the fact that a larger CSI error radius of ERs decrease the information signal quality, and it also increases the interference for ERs.

#### 5.5.3. Network Power Consumption versus ER Number

**Figure 7:**
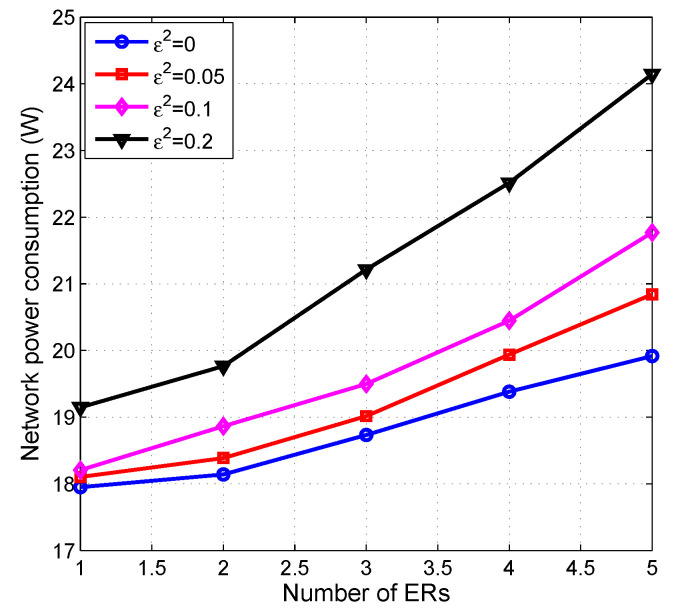
Network power consumption vs. number of ERs’ with γ=15 dB.

We also investigate the impact of ER number on network power consumption under different CSI error radius. The results are shown in Figure 7.

It can be seen from Figure 7 that the more overall network power that is consumed in order to interfere, the larger the number of ERs. This is mainly because more power is needed to guarantee all the SINR of ERs be no larger than their SINR thresholds. On the other hand, by increasing the channel error radius, more network power is consumed. This is because, with a higher CSI error $\varepsilon^{2}$, a larger amount of DTP and AN alongside the active BS number are required to maintain the SINR thresholds of ERs and IRs. In particular, when $\varepsilon^{2}=0$, the channels of IRs and ERs are perfectly known at the BBU pool. In this case, to support guaranteed QoS, the network power consumption is lower than the ones when the channels are imperfect.

## 6. Conclusions

This paper has investigated the power efficiency of a secure downlink C-RAN system with CSI uncertainty. The BS mode, beamforming, and AN are jointly optimized to minimize the overall network power consumption with imperfect CSI at both IRs and ERs. With problem transformation and approximation, a general IDC algorithm is proposed to provide a local optimal solution for the DC programming problem. A three-stage algorithm is proposed, which combines the IDC-based SDP algorithm and post processing method. Specifically, a rough sparse solution is obtained by the proposed IDC-based SDP algorithm, and the sparsity of the solution is further improved by a post processing procedure. Numerical results showed that the developed algorithm can significantly reduce the overall network power consumption. Moreover, by increasing the channel error or the number of ERs, more overall network power was consumed. The algorithm is developed under the assumption of imperfect CSI of ERs in this paper, and a more practical scenario without knowing the CSI of ERs is interesting, and we leave it for future work.

## Figures and Tables

**Table 1:** List of major symbols used in this paper.

Sym	Description	Sym	Description
Tr[·]	trace of a matrix	E[·]	expectation operator
$\Theta_{k}$	uncertainty channel shape of the ellipsoid of IR *k*	C	set of complex numbers
${\tilde{\Theta }}_{m}$	uncertainty channel shape of the ellipsoid of ER *m*	${\left(\cdot \right)}^{T}$	transpose
$H_{k}$	set of uncertainty channel region of IR *k*	${\left(\cdot \right)}^{H}$	conjugate transpose
$\Omega_{m}$	set of uncertainty channel region of ER *m*	$I_{N}$	N×N identity matrix
CN	complex gaussian distribution	$0_{N}$	N×N zero matrix
$P_{l}^{a,\mathrm{bs}}$	hardware power consumption of BS *l* in the active mode	$H^{N}$	N×N Hermite matrix
$P_{l}^{s,\mathrm{bs}}$	power consumption of BS *l* in the sleep mode	L	BS set
$P_{l}^{c,\mathrm{bs}}$	saved power by switching BS *l* in the sleep mode	A	active BS set
$P_{\mathrm{olt}}$	constant power consumption of OLT	Z	inactive BS set
$P_{l}^{a,\mathrm{tl}}$	power consumption of ONU *l* in the active mode	L∖Z	set of L exclude Z
$P_{l}^{s,\mathrm{tl}}$	power consumption of ONU *l* in the sleep mode	${∥x∥}_{0}$	$\ell_{0}$-norm
$P_{l}^{c}$	both BS *l* and its corresponding fronthaul link in active mode	${∥x∥}_{1}$	$\ell_{1}$-norm
$P_{l}^{a}$	both BS *l* and its corresponding fronthaul link in sleep mode	${∥x∥}_{2}$	$\ell_{2}$-norm
$P_{l}^{c}$	$P_{l}^{a}-P_{l}^{s}$	|·|	mode operator

**Table 2:** The transmit power distribution in milliwatt over eight random channels.

Ch	BS 1	BS 2	BS 3	BS 4	BS 5	BS 6	BS 7
Ch 1	0.19	0.09	0.07	0.07	0.13	1.59	0.23
Ch 2	8.58	2.22	3.19	1.02	2.76	12.32	2.18
Ch 3	4.09	2.55	2.76	4.85	12.56	7.71	1.16
Ch 4	2.08	0.70	1.68	3.44	2.63	1.58	2.64
Ch 5	4.39	12.68	2.19	0.37	1.67	2.18	14.49
Ch 6	7.99	1.52	6.88	7.93	1.01	1.13	0.52
Ch 7	4.14	5.79	1.99	0.09	2.57	3.64	0.86
Ch 8	1.50	10.27	3.68	0.22	2.99	0.44	2.70

**Table 3:** The active BS number, network power and transmit power consumed by different algorithms over Ch 2.

Algorithms	*A*	$P_{\mathrm{total}}$ (Watt)	$P_{\mathrm{tp}}$ (Watt)
Proposed algorithm	3	15.56	0.14
$\ell_{1}/\ell_{2}$-norm algorithm	4	20.28	0.07
ExSearch algorithm	3	15.56	0.14
Baseline algorithm	7	35.13	0.03

**Table 4:** The impact of BS antennas on network power consumption (Watt).

Algorithms	N=1	N=2	N=4	N=8
AN (Watt)	0.160	0.053	0.004	0.001
DTP (Watt)	0.317	0.134	0.013	0.008
NP (Watt)	27.43	18.18	15.32	12.79
AN/DTP(%)	50.50	39.55	30.77	12.50

**Table 5:** The impact of $\varepsilon_{ER}$ on power consumption (Watt) with $\varepsilon_{IR}^{2}=0.05$.

$\varepsilon_{\mathrm{ER}}^{2}$	0	0.001	0.01	0.05	0.1	0.12
AN + DTP	0.149	0.409	0.545	1.028	1.443	1.767
NP	15.599	17.553	18.191	18.998	19.971	20.929

## Appendix A. Proof of Proposition 1

**Proof.** Accordingly, constraint (23b) must hold with equality at the optimal point, i.e., $s_{l}^{★}=\sum_{k=1}^{K}\mathrm{Tr}\left(\Phi_{l}W_{k}^{★}\right)+\mathrm{Tr}\left(\Phi_{l}V^{★}\right)$, where $W_{k}^{★}$ and $V^{★}$ are respectively the optimal value of $W_{k}$ and V. Since each IR has its own QoS requirement, $W_{k}^{★}$ cannot be a zero matrix. Thus, $s_{l}^{★}\neq 0$ and the proof is completed. □
